# A Contemporary View of the Diagnosis of Osteoporosis in Patients With Axial Spondyloarthritis

**DOI:** 10.3389/fmed.2020.569449

**Published:** 2020-12-11

**Authors:** Mie Jin Lim, Kwi Young Kang

**Affiliations:** ^1^Division of Rheumatology, Department of Internal Medicine, College of Medicine, Inha University, Incheon, South Korea; ^2^Division of Rheumatology, Department of Internal Medicine, College of Medicine, The Catholic University of Korea, Seoul, South Korea; ^3^Division of Rheumatology, Department of Internal Medicine, College of Medicine, Incheon Saint Mary's Hospital, The Catholic University of Korea, Incheon, South Korea

**Keywords:** osteoporosis, axial spondyloarthritis, dual energy absorptiometry, trabecular bone score (TBS), quantitative computed tomography (QCT)

## Abstract

Axial spondyloarthritis (axSpA) is a chronic inflammatory disease that primarily affects the axial joints. Altered bone metabolism associated with chronic inflammation leads to both new bone formation in the spine and increased bone loss. It is known that patients with axSpA have a high prevalence of osteoporosis and fractures. However, there is no consensus on which imaging modality is the most appropriate for diagnosing osteoporosis in axSpA. Bone mineral density measurement using dual-energy X-ray absorptiometry is the primary diagnostic method for osteoporosis, but it has notable limitations in patients with axSpA. This method may lead to the overestimation of bone density in patients with axSpA because they often exhibit abnormal calcification of spinal ligaments or syndesmophytes. Therefore, the method may not provide adequate information about bone microarchitecture. These limitations result in the underdiagnosis of osteoporosis. Recently, new imaging techniques, such as high-resolution peripheral quantitative computed tomography, and trabecular bone score have been introduced for the evaluation of osteoporosis risk in patients with axSpA. In this review, we summarize the current knowledge regarding imaging techniques for diagnosing osteoporosis in patients with axSpA.

## Introduction

Osteoporosis is defined as a skeletal disorder characterized by compromised bone strength predisposing a person to an increased risk of fracture caused by minimal or low trauma. Bone strength was considered mainly dependent on bone density and quality (e.g., microarchitecture) (1). A deterioration in trabecular microarchitecture with a loss of connectivity between the trabeculae and cortical thinning is a typical trait of osteoporosis (2). Osteoporosis is a recognized entity in many inflammatory diseases. An increasing body of evidence indicates that current approaches for diagnosing osteoporosis are insufficient, and new methods are required to account for the different characteristics of chronic inflammatory arthritis.

Axial spondyloarthritis (axSpA) is one of the most common types of inflammatory arthritis and is known to be accompanied by a high prevalence of osteoporosis. AxSpA predominantly affects the axial skeleton, such as the sacroiliac joints and vertebrae. The term *axial spondyloarthritis* covers both patients with visible structural damage in the sacroiliac joints or spine, as seen on radiographs and categorized as ankylosing spondylitis (AS) or radiographic axSpA, and patients without such structural damage, categorized as non-radiographic axSpA (nr-axSpA) (3).

Bone disease in patients with axSpA is a complex phenomenon involving both bone loss and new bone formation, which impact the clinical features of the disease (4). Bone loss can occur locally, as erosions in the sacroiliac joints and vertebrae, or systemically, leading to an increased risk of osteoporosis and fracture (5). Patients with axSpA have a higher prevalence of both osteopenia and osteoporosis than age- and sex-matched controls (6). In axSpA, osteoporosis has multifactorial origins and can occur because of limited spinal mobility, increased proinflammatory cytokine levels, physical inactivity, or malabsorption (if inflammatory bowel disease is present) (5). Fragility fractures are a common outcome of osteoporosis. The high prevalence of osteoporosis in patients with axSpA leads to a higher risk of fracture (7). Therefore, it is important to assess the risk of osteoporosis in the early stages of axSpA and provide appropriate management.

Timely screening performed with dual-energy X-ray absorptiometry (DXA) is essential. Low bone mineral density (BMD) is a well-known feature of axSpA. The hallmarks of axSpA are sacroiliitis and spinal damage due to both bony erosion and abnormal bone formation. This can lead to the development of syndesmophytes, perivertebral bone formation, ankylosis of zygapophyseal joints, and pathological new bone formation in the ligamentous apparatus. This extensive osteoproliferation can make traditional DXA assessment of the spine in the anterior–posterior (AP) view, particularly in cases of structurally advanced disease, difficult to perform (4). This gives an illusion of a reassuringly normal BMD, even in cases in which osteoporosis may be present. Although not many axSpA patients show low BMD, bone structure and the quality of microarchitecture might be degraded in these patients (8). As patients with axSpA might still have poor bone health and fractures despite having a normal BMD, osteoporosis could be underestimated in such patients. There have been inconsistent reports on the association between low BMD and fractures in AS (9, 10). In axSpA patients with syndesmophytes, DXA of the hip or lateral spine can be performed (11). However, in such cases, DXA cannot assess bone quality. Increased fracture risk in axSpA patients is likely to be multifactorial, resulting from traditional osteoporosis risk factors and additional disease-related factors such as systemic inflammation, which affect not only BMD but also bone quality (12, 13). Therefore, including bone quality assessment when performing BMD measurements will enable a more accurate assessment of the risk of osteoporosis.

Recently, several new techniques to measure bone density or quality have been proposed to enhance fracture risk assessment in routine clinical practice. Some examples are trabecular bone score (TBS) and high-resolution peripheral quantitative computed tomography (HR-pQCT). TBS is a novel tool of estimating bone microarchitecture at the lumbar spine using DXA imaging. It is considered a non-invasive method for the assessment of trabecular microarchitecture (14). TBS has an additional advantage in that it is not affected by new bone formation, such as spinal osteophytes, which may lead to the overestimation of BMD in patients with lumbar spine osteoarthritis (15), as caused by syndesmophytes in patients with axSpA. HR-pQCT analyzes the trabecular and cortical compartments separately at the tibia and radius (16). It allows the measurement of large portions of distal bones with limited irradiation. HR-pQCT measures microarchitecture parameters, as well as volumetric density (16, 17).

Here, we review the appropriate methods for diagnosing osteoporosis in axSpA, focusing on recent studies on imaging methods used to assess bone impairment.

## Bone Mineral Density Assessment Using Dual-Energy X-ray Absorptiometry

DXA for BMD measurement is a standardized diagnostic tool for osteoporosis screening in patients with axSpA. Low bone mass, including osteopenia or osteoporosis according to the World Health Organization guidelines based on *T* scores (18), is frequently found in patients with axSpA (Table 1). Low bone mass is a well-known risk factor for vertebral fractures (20). There is a substantial increase in the risk of thoracolumbar compression fractures in patients with AS (32–34). Even AS patients with mild disease are at a higher risk of fractures than controls [odds ratio (OR), 5.92] (35). The risk of clinical spine fractures peaks in the first 2.5 years of AS, warranting early detection and treatment of low bone mass in these patients (34).

**Table 1:** Prevalence of low bone mineral density measured by DXA in patients with axSpA.

**References**	**Number (male/female)**	**Mean age (years)**	**Mean disease duration (years)**	**Prevalence of low BMD at AP lumbar spine**	**Prevalence of low BMD at the lateral lumbar spine**	**Prevalence of low BMD at the femoral neck**	**Prevalence of low BMD at the radius**
Kim HR et al. (19)	60 (51/9)	32.1	5.5	56%	NA	74%	NA
Wang et al. (20)	504 (417/87)	29.1	7.7	3% (OP only)	NA	9% (OP only)	1% (OP only)
Karberg et al. (21)	103 (66/37)	40.1	9.3	45%	NA	76%	NA
Malochet-Guinam et al. (22)	89 (52/37)	44.4	10.2	328%39% (in 28 females)	32.1% (in 28 females)	43%	NA
Toussirot et al. (23)	71 (49/22)	39.1	10.6	47%	NA	27%	NA
Kaya et al. (24)	55 (42/13)	35.8	11.05	56%	NA	55%	NA
Muntean et al. (25)	44 (44/0)	41	13.3	48%	NA	60%	NA
Klingberg E et al. (26)	204 (87/117)	50	15	21%25% (in females)	33% (in females)	29%24% (in females)	26% 21% (in females)
Meirelles et al. (27)	30 (27/3)	37	17	50%	NA	86%	NA
Korczowska et al. (28)	66 (66/0)	51.6	17.4	NA	NA	56%	68%
Speden et al. (29)	66 (0/66)	43.4	21.1	26%	NA	58%	NA
Deminger et al. (30)	168 (92/76)	55	24	10% (OP only)	NA	7% (OP only)	8% (OP only)
Magrey M. et al. (31)	100 (74/26)	46.1	83% of patients had >5 years	62% (≥50 years of age)	NA	41% (in patients ≥50 years of age)	NA

*BMD, bone mineral density; AxSpA, axial spondyloarthritis; DXA, dual-energy X-ray absorptiometry; AP, anterior–posterior; NA, not available; OP, osteoporosis*.

Lumbar spine DXA in the AP view includes both the vertebral body and posterior part of the vertebra, mainly consisting of dense cortical bone (36). Patients with nr-axSpA showed lower AP lumbar BMD values and *T* and *Z* scores than age- and sex-matched controls (37). A high frequency of vertebral fractures in patients with early spondyloarthritis (SpA) was associated with low BMD of the lumbar spine (38). A longitudinal study on early AS suggested that spine and hip BMD decrease in patients, especially during the active inflammatory stage (21, 39–41). Thirty-four early AS patients without ankylosis were followed up for 19 months; the follow-up lumbar spine and femoral neck BMD were reduced by 5% and 3%, respectively, in patients with active AS (42). However, patients with advanced AS frequently develop syndesmophyte and ligament ossification, resulting in false increases in AP lumbar BMD (25, 40, 43–47). When 168 patients with AS were followed up for 5 years, AP lumbar spine BMD increased, although femoral neck and radius BMD decreased (30). Thus, AP lumbar spine BMD is sensitive to bone loss in the early stages of the disease but not in the advanced stage. Therefore, alternative imaging techniques and parameters, including lateral spine or proximal femur BMD or QCT, are required for accurate diagnosis.

Lateral lumbar spine DXA evaluates the trabecular-rich vertebral body (36), making the technique less prone to the effects of new bone formation at the cortex. Lateral lumbar spine DXA is more sensitive than its AP counterpart in detecting low BMD in patients with AS (26, 46, 48, 49). An increase in the modified Stoke Ankylosing Spondylitis Spinal Score (mSASSS), the tool used to assess the presence of changes related to chronic AS, has been significantly correlated with a decrease in lateral lumbar spine BMD but not with AP lumbar spine BMD (26). Lateral spine BMD values were also significantly lower in AS patients belonging to a fracture group (46). Although the International Society for Clinical Densitometry guidelines currently do not recommend lateral lumbar spine DXA for the diagnosis of osteoporosis, they suggest that the technique be used to monitor the condition (50). Hence, lateral lumbar spine DXA could serve as a screening tool in late-stage AS patients with syndesmophytes (21).

Femoral neck BMD is sensitive to systemic bone loss in patients with axSpA. BMD at the femoral neck is reduced in patients with AS (40), and low bone mass, including osteopenia and osteoporosis, is significantly more common at the femoral neck than at the AP lumbar spine (19, 21, 22, 24, 25, 27–29, 31, 39, 43, 46). Prospective studies have shown that the BMD at the femoral neck decreased with the disease duration in patients with AS (24, 30, 31); such a decrease at a 2-year follow-up has been related to systemic inflammation, as demonstrated by elevated erythrocyte sedimentation rates (ESRs) (41). An increase in the mSASSS has been shown to be negatively correlated with femoral neck BMD (*r* = −0.324) and total hip BMD (*r* = −0.201) (26), suggesting that femoral neck BMD is less affected by new bone formation in AS. Low femoral neck BMD is also correlated with an increased risk of vertebral fracture (42, 51, 52), and AS patients with fractures display low femoral neck BMD (13, 46). In contrast to previous results in patients with axSpA, BMD and *T* and *Z* scores at the proximal femur in patients with nr-axSpA were similar to those in matched controls (37). Therefore, the best site to assess bone loss by DXA may be the femoral neck in patients with axSpA but not in patients with nr-axSpA.

Demineralization of the axial skeleton occurs in the early stages of AS, and as the disease progresses, the cortical bone of the peripheral skeleton also demineralizes (43). BMD at the radius in patients with AS tends to decrease during a 5-year follow-up (39), and patients with advanced AS (mean disease duration = 20.3 years) show depressed carpal BMD as compared to age-matched controls (53). Thus, for advanced stages of the disease, DXA of the wrist could prove a useful diagnostic tool.

## Trabecular Bone Score

TBS is a new method used to evaluate bone microarchitecture. It is a textural index that evaluates pixel gray-level variations in two-dimensional (2D) projection images of lumbar spine DXA scans, providing an indirect index of the trabecular microarchitecture of the lumbar spine (14). TBS obtained via a reanalysis of DXA scans is correlated with three-dimensional (3D) microarchitecture variables measured by QCT and HR-pQCT (54). Therefore, it may provide additional information on bone quality that cannot be captured by BMD measurement. The higher the TBS, the stronger the bone microarchitecture, which in turn leads to more resistance to fractures. A recent meta-analysis divided TBS into three groups based on fracture risk (55): normal, TBS ≥ 1.31; partially degraded, 1.31 > TBS > 1.23; degraded, TBS ≤1.23. TBS can predict osteoporotic fractures in postmenopausal women independent of the areal BMD of the hip or spine (56). Moreover, it is associated with hip fracture and major osteoporotic fracture risk in men older than 50 years (57). TBS has an advantage in evaluating osteoporosis in that it is not affected by spinal syndesmophytes, which may contribute to the overestimation of BMD in patients with axSpA (58).

Recently, several studies on TBS in patients with axSpA have been reported. Patients with axSpA had lower TBS than age- and sex-matched controls (59–61). However, the reported prevalence of low TBS varied widely, ranging from 22 to 56% (58–64) (Table 2). This variation may be associated with a difference in the applied patient-recruitment criteria [the Association of SpondyloArthritis International Society classification criteria for axSpA (65) or the New York classification criteria for AS (66)] and disease severity in the study patients.

**Table 2:** Prevalence of low trabecular bone score in patients with axSpA.

**References**	**Classification criteria**	**Number of patients (male/female)**	**Mean Age (years)**	**Mean disease duration (years)**	**Mean TBS**	Prevalence of low TBS^*^
Caparbo et al. (59)	AS	73 (73/0)	42	16	1.31	56%^*^
Kim et al. (62)	AS	54 (38/16)	40	8	1.37	41%#
Kang et al. (63)	AS	100 (100/0)	34	6	1.38	22%^*^
Wildberger et al. (58)	AxSpA	51 (51/0)	52	NA	1.26	NA
Hamoud et al. (60)	nr-AxSpA	60 (29/31)	35	NA	1.27	NA
Kang et al. (61)	AxSpA	248 (193/55)	39	10	1.38	22%^*^
Boussoualim et al. (64)	AxSpA; 65 pSpA; 4 Mixed; 26	95 (50/45)	41.1	8.4	1.34	46%#

*TBS, trabecular bone score; AxSpA, axial spondyloarthritis; pSpA, peripheral spondyloarthritis; nr-AxSpA, nonradiographic axial spondyloarthritis; NA, not available*.

* *Low TBS was defined as a score <1.31, #low TBS was defined as a score <1.35*.

TBS is not only associated with disease activity in patients with axSpA, as measured by inflammatory markers such as ESR and C-reactive protein and the Ankylosing Spondylitis Disease Activity Score (63, 64, 67), but is also negatively correlated with inflammation in patients with AS, as presented on lumbar spine magnetic resonance imaging (68). Additionally, we have reported a longitudinal association between disease activity measures and trabecular bone loss for 4 years in patients with axSpA (69). These findings mean that TBS might be a useful method for assessing osteoporosis risk related to inflammation in patients with axSpA.

A few studies on the association between TBS and the risk of fracture in axSpA have been reported (59, 63, 67, 70). Caparbo et al. studied 73 male AS patients and found an association between low TBS values and the prevalence of vertebral fractures. AS patients with low TBS (<1.310) tended to show higher frequencies of vertebral fracture (36.7 vs. 16.3%, *P* = 0.058) when compared with those with high TBS (≥1.310) (59). In a cross-sectional study of 255 patients with axSpA, we found that low TBS was associated with prevalent vertebral fracture, whereas lumbar spine BMD was not and that TBS showed better discriminatory values for prevalent vertebral fracture than total hip BMD (67). In addition, Richards et al. reported that baseline TBS independently predicted major osteoporotic and clinical vertebral fractures in 188 patients with AS, independent of Fracture Risk Assessment Tool scores (70). This finding suggests that TBS could be used as a useful method for incident fracture prediction.

Taken together, TBS derived from DXA images can be directly compared with BMD because both measure the same lumbar spine region. Not only does DXA enable fast and low-cost imaging, but it is also available in most clinical practice settings. Therefore, adding TBS assessment to BMD measurement can provide information about bone quality to detect bone impairment in patients with axSpA. TBS assessment is expected to be able to predict future fractures, as it can indirectly assess axSpA-induced changes in bone microstructure, beyond and independently of BMD.

## Quantitative Computed Tomography

Central QCT provides volumetric BMD (mg/cm^3^), as well as macrogeometry parameters at the level of the hip and spine (16, 71, 72). The bone geometry measurements from QCT are 3D parameters, whereas DXA-derived evaluations are extrapolated from 2D parameters (73). Several studies have performed hip or spinal QCT on patients with AS and assessed the results. Hip QCT was performed in 60 patients with AS and 57 healthy controls. Patients with AS had clinically lower areal BMD in the cortical bones and total bones of the proximal femur than healthy controls (74). In another study, 37 patients with AS were followed up for 10 years, and both DXA and QCT were performed at baseline and during the follow-up period (75). Spine QCT showed a statistically significant decrease in bone density, whereas spinal DXA showed an increasing trend in bone density. Correlation analyses performed between lumbar QCT and lumbar DXA found that the QCT trabecular volumetric BMD (vBMD) had the strongest correlation with DXA vBMD (*r*_S_ = 0.636; *P* < 0.001), followed by lateral lumbar spine BMD (*r*_S_ = 0.537; *P* < 0.001) and AP lumbar spine BMD (*r*_S_ = 0.380; *P* = 0.002) (75). QCT cortical vBMD was correlated with lateral DXA BMD (*r*_S_ = 0.595; *P* < 0.001), AP DXA BMD (*r*_S_ = 0.541; *P* = 0.002), and DXA vBMD (*r*_S_ = 0.431; *P* < 0.001) (16). Thus, QCT is more effective than lumbar spine DXA in revealing reduced BMD of the lumbar spine (76), although it has drawbacks of higher radiation doses and greater costs than DXA (16).

Single-energy QCT (SEQCT) offers the advantage of 3D evaluation of bone structure; Lange et al. reported a comparison of DXA and SEQCT in patients with AS (77). Patients were divided into four groups according to the degree of spine involvement. Both DXA and cortical bone measurement by SEQCT showed a decrease in bone density as spine involvement progressed and a sudden increase in bone density at the ankylosing stage. In contrast, trabecular analysis by SEQCT showed a gradual reduction in bone density as the disease progressed to the ankylosing stage (77). This study reflected two opposite trends of AS at the ankylosing stage: central trabecular bone loss and peripheral new bone formation of the spine, both characteristic features of AS. The disadvantage of SEQCT is that it significantly underestimates trabecular vBMD (depending on the actual vBMD, by up to 30%) when compared with dual-energy QCT (DEQCT), as the latter corrects for the effects of bone marrow fat (78). Although DEQCT has higher radiation exposure and variability than SEQCT, current scanners protect from radiation exposure and reduce variability to an acceptable range (78). Karberg et al. reported a satisfactory correlation between DEQCT at the spine and DXA at the femoral neck (21). Overall, low bone density was significantly more common at the DXA the femoral neck, followed by DEQCT and DXA at the lumbar spine. When patients were divided according to disease duration osteoporosis in patients with early AS (disease duration <5 years) was detected by DXA at the lumbar spine and femoral neck in 15% and 11% of patients, respectively, and no patients were found to be have osteoporosis on DEQCT (21). In contrast, the proportion of osteoporotic patients with long-standing AS (disease duration >10 years) assessed by DXA at the lumbar spine and femoral neck was 4 and 29%, respectively. Moreover, 18% of the patients with long-standing AS (disease duration >10 years) were found to have osteoporosis by DEQCT (21). The likelihood of finding syndesmophytes increased as the disease progressed. In patients with syndesmophytes, the frequency of low bone density was higher as measured by DXA at the femoral neck or DEQCT than by lumbar spine DXA (21). Therefore, osteoporosis was more frequently detected in patients with syndesmophytes and long disease duration, when measured by DXA at the femoral neck and DEQCT.

## High-Resolution Peripheral Quantitative Computed Tomography

HR-pQCT has been gaining attention as an alternative modality by which to assess BMD. It is distinguished from QCT by the creation of a high-resolution image, allowing for examination of the bone trabecular and cortical microstructure, an essential correlate of bone strength. This technology calculates BMD, cortical BMD, and trabecular BMD simultaneously while exposing the patient to a far lower dose of radiation than in typical computed tomography (CT) (79, 80). HR-pQCT measures the trabecular and cortical compartments separately at the tibia and radius, allowing measurement of large portions of distal bones with good spatial resolution and minimal irradiation (16, 17). HR-pQCT measures microarchitecture parameters such as trabecular thickness, number and distribution, and cortical porosity of the tibia and radius, thus resembling a virtual bone biopsy of the peripheral bone (16, 79, 81, 82). It was previously argued that osteoporosis in patients with axSpA primarily affects the axial skeleton (23, 83). HR-pQCT helps reveal poor microarchitecture of the trabecular and cortical bones in the peripheral skeleton of patients with AS. Klingberg et al. performed HR-pQCT, QCT, and DXA of the lumbar spine in 69 male patients with AS and healthy controls and successfully compared these three bone-analyzing techniques (13). HR-pQCT of the radius and tibia showed lower vBMDs both in the cortical bone of the radius and in the trabecular bone of the tibia in patients with AS than in controls (13). Low lumbar trabecular vBMD measured by QCT significantly correlated with poor bone microarchitecture indices measured by HR-pQCT, such as thinner trabecula, lower trabecular number, thinner cortex, lower cortical volumetric BMD, and increased cortical porosity (13). AS patients with a vertebral fracture had substantially lower cortical lumbar vBMD as measured by QCT and lower BMDs as measured by DXA at the hip, AP, and lateral lumbar projection than age-matched AS controls without fractures (13). HR-pQCT also displayed significantly lower trabecular and cortical vBMDs in the radius and lower trabecular thickness, cortical thickness, and cross-sectional area in both the radius and tibia in AS patients with fractures than in the age-matched AS controls without fractures (13). Increases in mSASSS correlated significantly with decreases in trabecular vBMD in the lumbar spine by QCT (*r*_s_ = −0.620, *P* < 0.001), increases in cortical porosity (*r*_s_ = 0.352, *P* = 0.004 in the radius; *r*_s_ = 0.363, *P* = 0.002 in the tibia), and decreases in trabecular thickness (*r*_s_ = −0.528, *P* < 0.001 in the radius; *r*_s_ = −0.488, *P* < 0.001 in the tibia) and vBMD of the trabecular and cortical bone (*r*_s_ = −0.4, *P* = 0.001 in the radius; *r*_s_ = −0.475, *P* < 0.001 in the tibia) as measured by HR-pQCT (13). With the existence of syndesmophyte as the binary outcome, decreasing lumbar trabecular vBMD [*B* = −0.058; *P* < 0.001; OR = 0.943; 95% confidence interval (CI), 0.917–0.970] and increasing lumbar cortical vBMD (*B* = 0.019; *P* = 0.016; OR, 1.019; 95% CI, 1.004–1.035) were independently associated with syndesmophyte formation (13). In another study by Neumann et al., HR-pQCT showed pronounced bone loss in the cortical area, cortical thickness, and cortical BMD in patients with nr-axSpA as compared to controls. However, trabecular vBMD did not differ between patients and controls (81). Patients with short disease duration (<2 years) also showed a significant reduction in cortical thickness and cortical area when compared with controls, and the decrease in cortical thickness was more prominent in long-term patients (disease duration >2 years). Therefore, bone loss in the cortical bone probably develops in the early stages of SpA. HR-pQCT aids in the identification of many structural and compartmental changes in bone tissue in patients with axSpA.

## Finite Element Analysis in Peripheral Quantitative Computed Tomography

Finite element analysis (FEA) is an approach that has been established as useful in describing bone quality. FEA is a standard method for bone fragility determination both *in vivo* and *in vitro*. Finite element–based simulation models integrate both bone quantity and quality. FEA can assess bone fragility directly from the bone mass distribution, material behavior of the bone extracellular matrix, and classic mechanics principles (84). High-quality CT, including QCT and HR-pQCT, allows finite element modeling. Finite element models for bones may be divided into two groups: micro-finite element (μFE) models, in which the trabecular and cortical bone morphology is modeled in detail (85, 86), and homogenized, continuum-level (hFE) models, in which one element covers a wider bone area, which is considered as homogeneous material (87, 88). Although both models have unique strengths and weaknesses, μFE models are highly accurate, and they are considered to be the gold standard for bone models (84). μFE models, which are usually based on HR-pQCT of the proximal tibia and distal radius, lay out the trabecular and cortical bone morphology in detail (79), whereas hFE models, based on QCT images, do not achieve the same levels of detail (84). Several studies have assessed bone fragility in patients with AS using FEA of HR-pQCT data (8, 59). Patients with AS exhibited lower values for bone strength parameters of the distal tibia (59) and distal radius (8) than healthy controls. Besides assessing bone quality in patients with AS, FEA also provides a biomechanical model that enables creation of a 3D model of the AS kyphotic spine (89) and simulation of the effects of surgical implants on AS-related spinal fractures (90). These findings show that FEA could be used to evaluate bone fragility in patients with axSpA.

## Conclusion

We reviewed current literature regarding imaging techniques for diagnosing osteoporosis in patients with axSpA, including more recent modalities for assessing bone quality. An increasing body of evidence shows that the inclusion of bone quality assessment by using other modalities (e.g., TBS or QCT) in traditional evaluation of BMD is essential for osteoporosis risk assessment in patients with axSpA. Future studies should focus on whether these specific imaging techniques for optimizing the diagnosis and management of osteoporosis can decrease the incidence of new fractures in patients with axSpA.

## Author Contributions

ML and KK conceptualized the review and wrote the original draft of the paper. ML acquired funding. KK reviewed and edited the manuscript. All authors contributed to the article and approved the submitted version.

## Conflict of Interest

The authors declare that the research was conducted in the absence of any commercial or financial relationships that could be construed as a potential conflict of interest.
